# Decision Support System for Predicting Survivability of Hepatitis Patients

**DOI:** 10.3389/fpubh.2022.862497

**Published:** 2022-04-14

**Authors:** Fahad R. Albogamy, Junaid Asghar, Fazli Subhan, Muhammad Zubair Asghar, Mabrook S. Al-Rakhami, Aurangzeb Khan, Haidawati Mohamad Nasir, Mohd Khairil Rahmat, Muhammad Mansoor Alam, Adidah Lajis, Mazliham Mohd Su'ud

**Affiliations:** ^1^Computer Sciences Program, Turabah University College, Taif University, Taif, Saudi Arabia; ^2^Faculty of Pharmacy, Gomal University, Dera Ismail Khan, Pakistan; ^3^Faculty of Engineering and Computer Sciences, National University of Modern Languages-NUML, Islamabad, Pakistan; ^4^Faculty of Computer and Information, Multimedia University, Kuala Lumpur, Malaysia; ^5^Center for Research and Innovation, CoRI, Universiti Kuala Lumpur, Kuala Lumpur, Malaysia; ^6^Institute of Computing and Information Technology, Gomal University, Dera Ismail Khan, Pakistan; ^7^Division of Pervasive and Mobile Computing, Information Systems Department, College of Computer and Information Sciences, King Saud University, Riyadh, Saudi Arabia; ^8^Department of Computer Science, University of Science and Technology, Bannu, Pakistan; ^9^Faculty of Computing, Riphah International University, Islamabad, Pakistan; ^10^Malaysian Institute of Information Technology, University of Kuala Lumpur, Kuala Lumpur, Malaysia; ^11^Faculty of Computing and Informatics, Multimedia University, Cyberjaya, Malaysia; ^12^Faculty of Engineering and Information Technology, School of Computer Science, University of Technology Sydney, Ultimo, NSW, Australia

**Keywords:** disease diagnosis, deep learning, hepatitis diagnostics, decision support system, bidirectional LSTM

## Abstract

**Background and Objective:**

Viral hepatitis is a major public health concern on a global scale. It predominantly affects the world's least developed countries. The most endemic regions are resource constrained, with a low human development index. Chronic hepatitis can lead to cirrhosis, liver failure, cancer and eventually death. Early diagnosis and treatment of hepatitis infection can help to reduce disease burden and transmission to those at risk of infection or reinfection. Screening is critical for meeting the WHO's 2030 targets. Consequently, automated systems for the reliable prediction of hepatitis illness. When applied to the prediction of hepatitis using imbalanced datasets from testing, machine learning (ML) classifiers and known methodologies for encoding categorical data have demonstrated a wide range of unexpected results. Early research also made use of an artificial neural network to identify features without first gaining a thorough understanding of the sequence data.

**Methods:**

To help in accurate binary classification of diagnosis (survivability or mortality) in patients with severe hepatitis, this paper suggests a deep learning-based decision support system (DSS) that makes use of bidirectional long/short-term memory (BiLSTM). Balanced data was utilized to predict hepatitis using the BiLSTM model.

**Results:**

In contrast to previous investigations, the trial results of this suggested model were encouraging: 95.08% accuracy, 94% precision, 93% recall, and a 93% F1-score.

**Conclusions:**

In the field of hepatitis detection, the use of a BiLSTM model for classification is better than current methods by a significant margin in terms of improved accuracy.

## Introduction

Data mining is becoming more common in a variety of industries, including business, education, and healthcare, as a result of the rise of AI. Many researchers are interested in healthcare decision support systems because they make it possible to glean valuable information from vast amounts of medical records. Decision support systems may be able to speed up the diagnosis process for human health care providers (1). Data mining techniques known as “deep learning” (DL) employ a variety of feature-encoding algorithms to learn from past data and deliver accurate predictions (2). Sentiment categorization (3), smart agriculture (4), and other applications are among them. In the last several years, neural network models have shown a stunning capacity to predict and classify information. Deep learning algorithms, as demonstrated by Shickel et al. (5) and Miotto et al. (6), have appeared notably in healthcare for knowledge discovery and illness diagnosis, such as heart diseases, mental disorders, and hepatitis disorders, utilizing health data.

### A Need for Hepatitis Disease Prediction

Viral hepatitis is a major public health concern around the world. Hepatitis A and E viruses (HAV and HEV) are endemic in many low-income countries. They usually cause self-limiting hepatitis, but in case of compromised liver status, they can cause fulminant liver failure and chronic infection. Hepatitis B and C viruses (HBV and HCV) both cause acute liver injury and are more frequently associated with progressive liver fibrosis, cirrhosis, and a higher likelihood of liver cancer (hepatocellular carcinoma or HCC). Hepatitis D virus (HDV) is often transmitted by co-infection with HBV. Because of the severity of complications, co-infection is considered the most serious type of viral hepatitis. Hepatitis viruses are 100 times more infectious than HIV/AIDS and are the leading cause of liver cancer, which is the world's second-leading cause of cancer-related deaths. Abnormalities in liver function tests such as aspartate aminotransferase, alanine aminotransferase, alkaline phosphatase, gamma-glutamyl transferase, lactate dehydrogenase, bilirubin, and albumin can indicate the liver injury and are the integral part of the biochemical investigations in hepatitis patients. Hepatitis is one of the leading causes of liver cancer, which underscores the critical importance of early detection and diagnosis for an effective antiviral treatment (7–10).

### Aims of the Project

Computational techniques like machine learning (ML) have been researched by a majority of researchers (8, 9) in recent years in order to predict the prevalence of hepatitis. Detecting hepatitis before symptoms manifested was the primary focus of their scientific work, and they were successful in this aim. Conventional encoders couldn't handle the illness dataset's predictors' relationships well. For the sake of this investigation, the suggested BiLSTM-based framework properly identifies (survivability or death) in patients with severe hepatitis. The goal of this research is to develop a prediction model that will assist physicians in determining the prognosis and survival of hepatitis patients based on medical conditions provided by the patients themselves. As a result, patients who receive early care are less likely to die.

### Baselines

Akbar et al. (9) created an ML-based hepatitis disease prediction method. ML was used to better predict hepatitis using random forest (RF), support vector machine (SVM), and other classifiers. There are intrinsic links between the predictors, but this is not taken into consideration by ML classifiers. Because of this, current ML models fail to accurately forecast hepatitis patients' long-term survival using medical information. BiLSTM is an updated DL model that has previously been applied effectively in several areas, such as DDoS attack prediction (10), behavior identification (2), and others (3). To better understand hepatitis, we created a BiLSTM model. The steps are as follows: an oversampling method and a BiLSTM model were used to balance the dataset for hepatitis. Using our recommended technique, we can anticipate hepatitis patient survival from patient health data by using both data balance and the BiLSTM layer.

### Problem Statement

It is challenging to reliably predict hepatitis from patient data when standard feature sets are used in conjunction with a machine learning classifier (8, 9). Aside from that, the absence of important information makes DL models for the prediction of hepatitis sickness less successful than they could be. To deal with the aforementioned challenges, the prediction of hepatitis from patient data is considered a binary-label prediction problem. The presence of hepatitis is predicted based on the disease dataset provided. The dataset contains two types of class labels: D1 (Hepatitis_patient_ survivability = yes) and D2 (Hepatitis_patient_ survivability = no). In order to predict whether or not someone has hepatitis, the neural network makes use of these two sets of information: we intend to use a deep neural network to construct an automated system that can learn from training data and predict the survivability of hepatitis patient based on contextual information.

### Research Questions

The following are the research questions we hope to achieve in our study so that we can accurately diagnose hepatitis.

RO1: Using patient sickness data, the BiLSTM deep learning model will be used to create predictions regarding hepatitis infection.
RO2: BiLSTM model's comparison with traditional machine learning and deep learning models for hepatitis prediction.
RO3: The efficiency of the suggested technique in predicting hepatitis patients is compared to previous studies.

### Research Contributions

The following are some of the most significant outcomes of this research work:

1. The development of a deep learning (BiLSTM) system for hepatitis disease diagnosis.
2. The proposed deep learning hepatitis detection approach outperforms existing machine learning techniques.
3. The effectiveness of the model in predicting hepatitis has been significantly improved as a result of the recommended strategy.

The following is the sequence in which the remaining sections of the study are organized: Section Related Work provides a review of the existing literature, and section Proposed Methodology outlines the approach that has been proposed by the authors. section Experimental Results and Discussion presents the findings and discussion, and Section Conclusion and Future Work discusses the application of the suggested strategy in the future.

## Related Work

This section summarizes previous research on hepatitis illness prediction.

Chicco and Jurman (7) used a RF classifier model to examine the EHRs of 540 control subjects and approximately 80 patients who had been diagnosed with hepatitis C. We use the best classifier (Random Forests) to find the most important variables for hepatitis C. The work of Kashif et al. (8) focuses on predicting LOLA therapy responses in hepatitis c patients. They employed K Nearest Neighbor, kStar, Bayesian Network, Randomized Forest, Radial Basis, PART, Logistic Regression, OneR, Svms, and Multi-Layer Perceptron to estimate therapeutic efficacy. For the purpose of evaluating the effectiveness of machine learning algorithms, several performance metrics are employed. It was shown that CDSS may be used to predict and diagnose Hepatitis-B infection in studies by Panigrahi et al. (11). The expert system's knowledge base has 59 rules. ES-builder, a web-based Expert System Shell, is used to implement the envisioned system (ESS). Querying the suggested system has been extensively tested to verify its efficacy. The goal of the study conducted by Wicaskno and Mudiono (12) is to use artificial intelligence to help diagnose hepatitis effectively. In this study, the certainty factor was used to diagnose the kind of hepatitis early on. Based on the data provided by the patients, this study's findings will aid in the early diagnosis of hepatitis types with precision. In order to forecast HBV integration sites, Wu et al. (13) created the DeepHBV model. DeepHBV was taught and evaluated using HBV inclusion data files from the dsVIS database by autonomously learning localized genomics features. By combining machine learning and artificial neural networks, Butt et al. (14) created an Intelligent Hepatitis C Stage Diagnosis System (IHSDS) (ANN). Only 19 of 29 attributes from the Uci repository were chosen for the study; 70% of this dataset was used for training and 30% for validation. Orooji and Kermani (15) employed machine learning to handle unbalanced data in hepatitis diagnostics. They used data preparation, data analysis, examination, and software implementation as phases. Data balance strategies were used in data preprocessing. When it comes to predicting the diagnosis of individuals with persistent hepatitis, Parisi and RaviChandran (16) have developed an innovative variable selection (FS) technique that merges neighborhood component analysis and ReliefF by taking the average of their findings, paired with a Lagrangian SVM Classifier (LSVM), which is utilized as an ML-based classification model for decision-making. Wu et al. (17) published the results of a comprehensive review and meta-analysis of the available literature, followed by validation and attention in an external cross-section of 986 cases. The area under the curve method (AUROC) and the calibrating indices were used to evaluate the model's potential to anticipate HCC within three, five, seven, and ten years. Table 1 presents a review of selected studies.

**Table 1:** A review of selected studies.

**Study**	**Technique(s)**	**Results**	**Limitations**
Chicco and Jurman (7)	RF classifier model	The [−1, +1] interval of MCC has increased by 8.25%	The validation cohort dataset lacked some of the attributes of the discovery cohort dataset.
Kashif et al. (8)	K Nearest Neighbor, kStar, Bayesian Network, Randomized Forest, Radial Basis, PART, Logistic Regression, OneR, Svms, and Multi-Layer Perceptron	Acc: 87%	Lack of data balancing techniques
Panigrahi et al. (11)	Web-based Expert System Shell	Knowledge base consists of 59 rules to design the expert system	Procedural knowledge can be enhanced for more effective diagnosis
Wicaksno and Mudiono (12)	certainty factor was used for early diagnosis of hepatitis	CF = 97%	Limited rule base
Wu et al. (13)	DeepHBV model	AUROC = 0.6363 AUPR = 0.5471	Lack of appropriate hidden layer selection
Butt et al. (14)	Intelligent Hepatitis C Stage Diagnosis System	Precision (94%)	Lack of external validation
Orooji and Kermani (15)	machine learning to handle unbalanced data in hepatitis diagnostics	More than 90%	Skewed dataset
Parisi and RaviChandran (16)	Merges neighborhood component analysis and ReliefF Lagrangian SVM Classifier (LSVM)	F1-score = 94%	Expanding its applicability to additional hematological diseases in order to improve patient outcomes more comprehensively.

## Proposed Methodology

**Figure 1:**
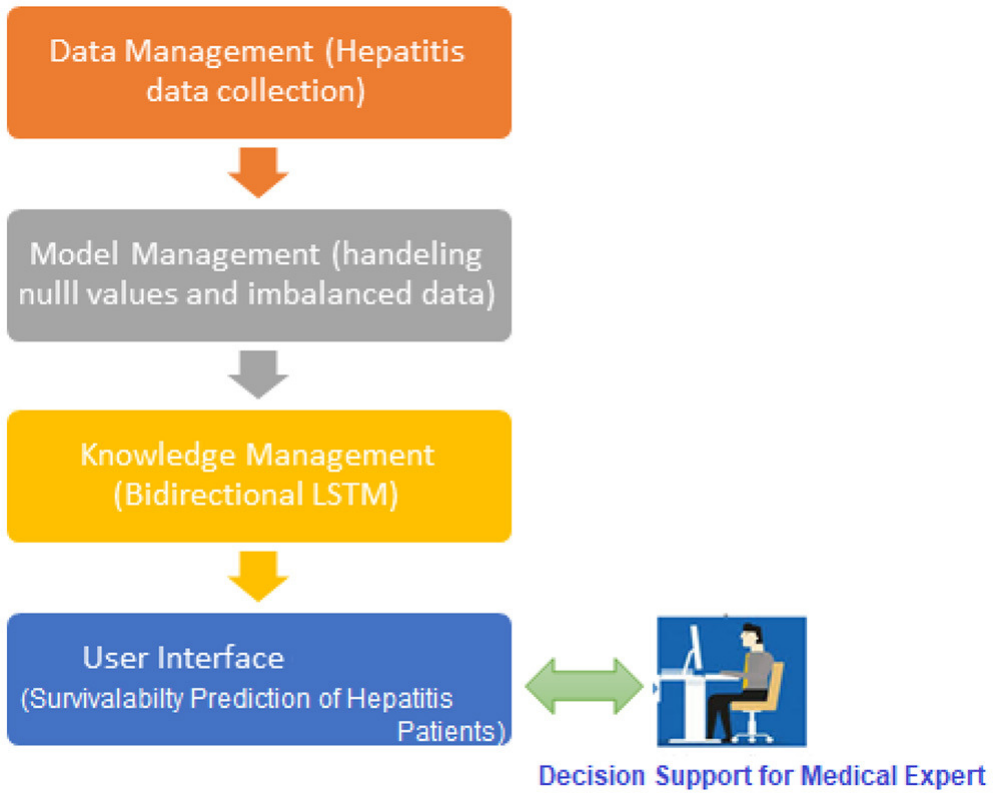
A preview of the proposed hepatitis disease prediction system.

For this complex problem of decision-making, it is critical to use deep learning techniques to combine existing data and experience into a DSS. Our DSS (see Figure 1) incorporates data, experience, and models so that hepatitis clinicians may use this knowledge to form diagnostic decisions. As recommended by Turban et al., we spoke with medical experts at every phase of the design process, in order to include the users even more deeply. We followed Turban et al. (1) in building the DSS because of the DSS's nature and the sophistication of the decision topic. There are four major components of the DSS: data management, model management, knowledge-based management, and user interface.

### Data Management System

**Figure 2:**
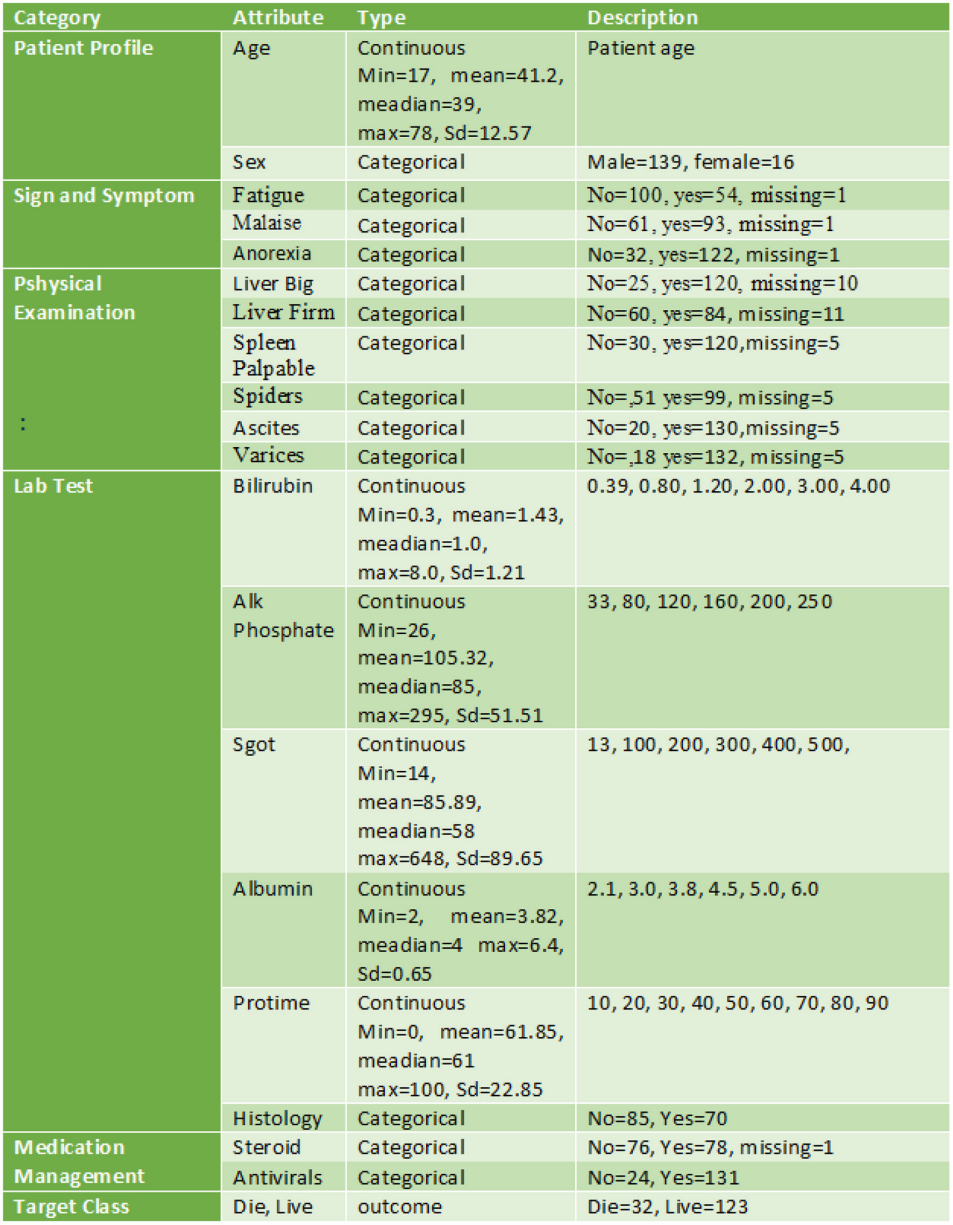
Hepatitis dataset breakup.

A decision support system (DSS) makes use of databases and/or datasets in order to give relevant data to the decision support system. In addition to local, public, and customized sources (1), DSS data may be obtained from organizational and official sources (1). The dataset utilized in this study is the Hepatitis dataset from the University of California, Irvine repository (18). If we look at the dataset (see Figure 2), there are 155 instances with 20 attributes. The dataset contains 19 attributes and a single class (outcome), which may be divided into the following five groups (19).

#### How to Make Use of the Data

The spreadsheets are transformed into CSV format. The “csv” command line option is specified via the “pd.read” command—line interface option. This is an important Panda utility. We divided the training/testing sets through using sklearn train (80%) and test (20%) partitioning tool (2) to get the desired results.

#### Train Set

Approximately 80% of the data from the training set was used throughout the training process (3). Both outcome descriptors (response variables) and input parameters are included in the training phase (predictor variables).

#### Validation Set

Overfitting and underfitting may be handled by using validation data in the system. The model is evaluated using data from a 10% validation subset (2). When working with Keras, you have the option of changing parameters manually or automatically. Automated validation in this study will allow for a more objective evaluation of the suggested strategy.

#### Test Set

The test set offers unique cases to assess the algorithm's effectiveness. This process is used after considerable training and testing. Evaluation of the model is possible through the use of a test set (10). 10% of the testing data was employed, with no relation to the training instances. It is utilized once the model has already been adequately trained. It is then validated against actual data. Scikit-train-test divides the data into 90/10 ratios, with 10% of the data used for testing and the other 90% used for learning. Using a validation set, we adjusted the model's settings and analyzed the outcomes.

#### Treatment of Data

A 10-fold cross-validation test is used to verify the model. Ten copies of the training instance are collected and stored at each level. In this instance, we looked at one more “holdout” model. The variant with the greatest F1 score was selected for the holdout sample.

### Model Management System

It performs the function of a data management system. Incorporated into DSS is a modelbase of statistical and other algorithms that allow for the provision of complex analytics. An MMS transforms data from a DMS into information by applying models to it. For the purpose of developing a viable prediction model, it is necessary to adequately pretreat the available health data. Null value substitution and imbalanced datasets are both taken care of by the data management system.

#### Management of Unbalanced Data Sets

There is a considerable imbalance in the underlying dataset, which treats both groups unequally. Patients who die make up 26% of the class, whereas 74% of those who live make up the remaining 26% of the class. It might be difficult to get accurate predictions from predictive algorithms when the data is imbalanced.

When a model learns from data that is skewed and unequally classified, the output generally favors the main class while neglecting the smaller categories during the classification step (20). This is seen as a class imbalance issue. Oversampling, a data processing sampling technique, is used to equalize all instances of a class. Small classes can be expanded by oversampling. Increasing the imbalance percentage is all that random up sampling accomplishes. The categorization results were much improved with the addition of small group replication. In both the T1:415 and T2:414 classes, random oversampling is applied to the balanced dataset in the same way. The total number of instances is 829.

#### How to Deal With Missing Data

Reliable data should be entered into the model to patch in any empty attributes (20). Filling the spaces can be done by averaging the values of the items in the previous levels, picking a random element, or switching back and using the previous level's number. Following our decision to go with the third alternative, we proceeded to update all of the missing data. In the dataset, there are just a few missing values. The PROTIME attribute has 67 missing values (43%), accounting for <10% of the total dataset.

### The Knowledge Management System

**Figure 3:**
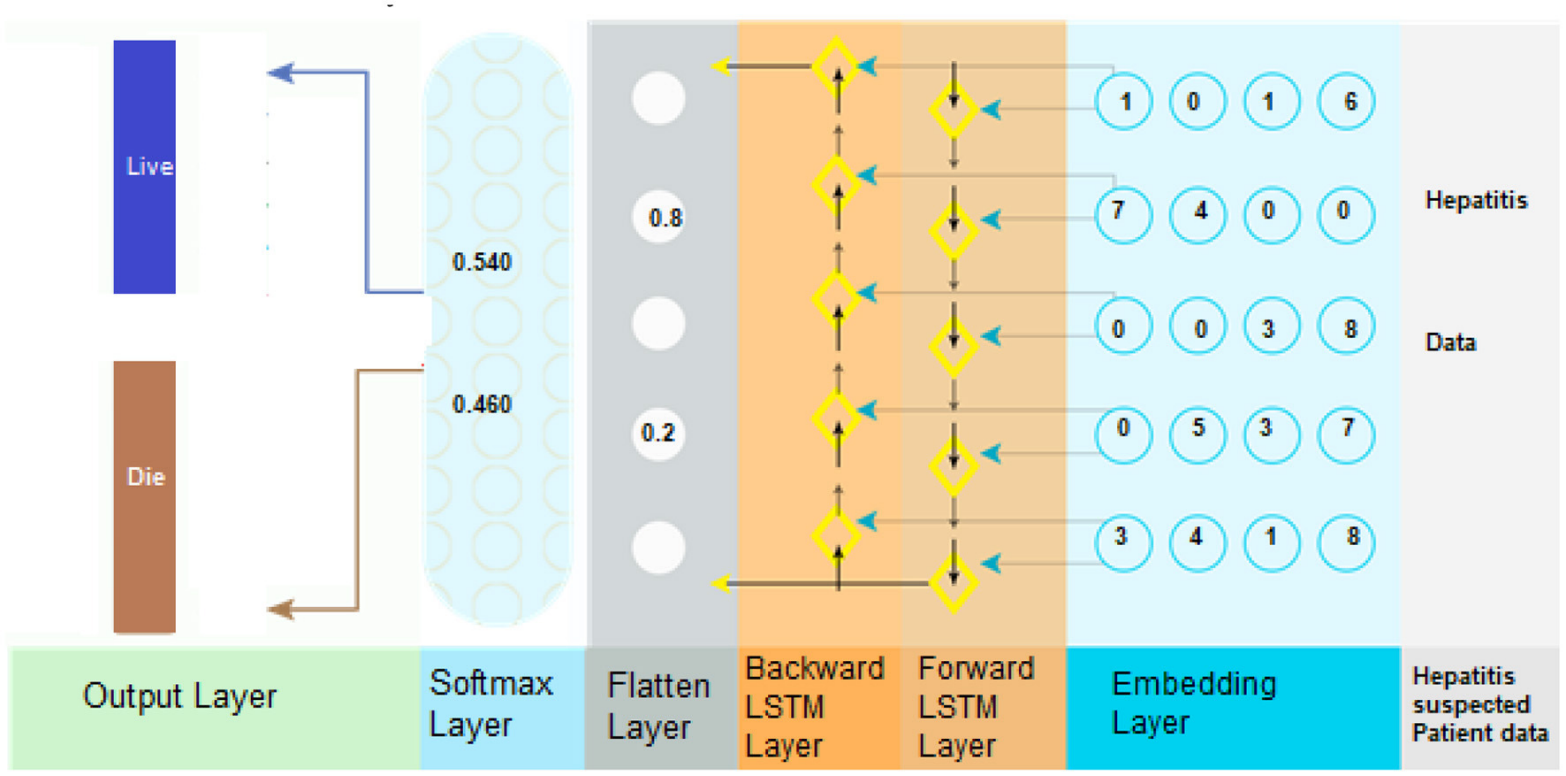
BiLSTM is used for the hepatitis prediction system.

Combined with other DSS modules, this component may be able to offer the most current information to help fix the issue. It is necessary to change information into knowledge when it has been discovered, acquired, and organized in some way. Analyzing and categorizing data is an essential part of the research process. To summarize, the suggested model consists of three main parts: embedding layer data structure, storing forward as well as backward contextual information, and softmax layer-based categorization. The second module can encapsulate features using numeric representation (21). Using Bi-LSTM to encode data contexts inside a sequence when it comes time for the final module, softmax activation is used to classify the data (see Figure 3). Here's a breakdown of each component:

#### Embedding Layer

Using embedding, it is possible to convert items (categories) into a continuous numeric array of a certain size (vectors). In neural networks, elements represent discrete variables as low-dimensional vector embeddings, which are then represented as a function of the input data. There are a number of advantages to utilizing neural embeddings in categorical data reduction and in identifying categories in the resultant space over other methods of data reduction and description. Keras facilitates the usage of embeddings by making them more accessible. Embedding vectors with attribute-level representations were employed to represent the data in the hepatitis dataset, with an embedding dimension that was equal to or greater than the dimension of the input data (22). The data embedding vector was constructed using a Keras embedding layer with 32 layers. Within the embedding layer, a two-dimensional embedding matrix (feature matrix) was built in which “represents the length of the input data” and “denotes the dimension of a word embedding” were used as input parameters. Once the embedding matrix had been produced, it was moved to the next tier in the hierarchy.

#### The Bi-Directional LSTM Layer

When predicting hepatitis sickness, the suggested technique makes use of a deep neural network, especially a bidirectional long-short time memory (BiLSTM), which can distinguish between D1 (Hepatitis_patient_ survivability = yes) and D2 (Hepatitis_patient_ survivability = no). Long-term dependencies are discovered through the use of the Bi-LSTM layer. As a result, it aids in the preservation of the two preceding and subsequent contexts of encoded data (23). In place of storing information from earlier contexts, a single unidirectional LSTM simply retains the information that has already been stored. As a result, Bi-LSTM is capable of analyzing encoded patient data in considerably more depth. Bi-LSTM employs both forward and backward LSTM to learn the context of data in the past and future (2). This is accomplished by the use of the following mathematical formulas (Equations 1–12).

The formulas for Forward LSTM:

(1) $$\begin{array}{r} f_{t}=\sigma \left(W_{f}x_{t}+U_{f}h_{t-1}+b_{f}\right) \end{array}$$

(2) $$\begin{array}{r} i_{t}=\sigma \left(W_{i}x_{t}+U_{i}h_{t-1}+b_{i}\right) \end{array}$$

(3) $$\begin{array}{r} o_{t}=\sigma \left(W_{o}x_{t}+U_{o}h_{t-1}+b_{o}\right) \end{array}$$

(4) $$\begin{array}{r} c\sim t=\tau \left(W_{c}x_{t}+U_{c}h_{t-1}+b_{c}\right) \end{array}$$

(5) $$\begin{array}{r} c_{t}=f_{t}⊙c_{t-1}+i_{t}⊙{c\sim }_{t} \end{array}$$

(6) $$\begin{array}{r} h_{t}=o_{t}⊙\tau \left(c_{t}\right) \end{array}$$

The formulas for Backward LSTM:

(7) $$\begin{array}{r} f_{t}=\sigma \left(W_{f}x_{t}+U_{f}h_{t+1}+b_{f}\right) \end{array}$$

(8) $$\begin{array}{r} i_{t}=\sigma \left(W_{i}x_{t}+U_{i}h_{t+1}+b_{i}\right) \end{array}$$

(9) $$\begin{array}{r} o_{t}=\sigma \left(W_{o}x_{t}+U_{o}h_{t+1}+b_{o}\right) \end{array}$$

(10) $$\begin{array}{r} c\sim t=\tau \left(W_{c}x_{t}+U_{c}h_{t+1}+b_{c}\right) \end{array}$$

(11) $$\begin{array}{r} c_{t}=f_{t}⊙c_{t+1}+i_{t}⊙{c\sim }_{t} \end{array}$$

(12) $$\begin{array}{r} h_{t}=o_{t}⊙\tau \left(c_{t}\right) \end{array}$$

#### Prediction Based on the SoftMax Algorithm

After that, SoftMax is utilized to determine the probability of forecasting target labels (i.e., the hepatitis disease). Using the formula (Equation 13), it is possible to calculate the net value of the input.

(13) $$\begin{array}{r} d_{i}=\;\sum w_{i}l_{i}+b \end{array}$$

The input vector is denoted by “l,” whereas the weight vector is denoted by “w.” Bias is represented by “b.” By putting it into Equation (14), one can calculate the SoftMax.

(14) $$\begin{array}{r} softmax\;\left(d_{i}\right)=\frac{expd^{d_{i}}}{\sum_{n=1}^{m}\exp^{d_{n}}} \end{array}$$

### Applicable Case

Based on the available illness data, we ran a number of computations to predict the presence of hepatitis. Every level of the BiLSTM model is described in depth.

#### Preparing the Data

For each patient occurrence in the disease dataset, our model predicts whether the patient has hepatitis D1: hepatitis Yes or “D2: hepatitis No.”. Data on disease is first obtained for the DL model by using the attribute selection module. A Keras parser was used to turn the input into a matrix of indexes, which was then sent to the embedding layer of the DL model for assessment. Using the embedding layer, each illness indication is transformed into a vector of streaming values. [0.6 0.4 0.3 0.7] is an example of a vector embedding, which contains information about disease in the form of the index [1]. It ended out like this in terms of the wrapping of the matrices: [0.66, 0.53, 0.75, 0.14], [0.62, 0.71, 0.41], [0.81, 0.42, 0.74, 67], [0.54, 0.28, 0.41].

#### Retrieval of Contextual Data

A corrected feature map created from the previous deep network serves as input for this layer's neural network. The most important components in BiLSTM layer computations are the potential candidate value (*c*~_*t*_), output gate (*o*_*t*_), forget gate (*f*_*t*_), and input gate (*i*_*t*_).

#### 1st Hidden Layer

**Figure 4:**
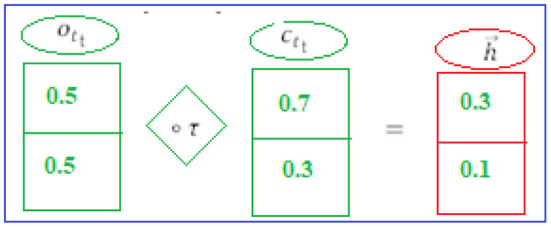
1st hidden layer.

(*i*_*t*_) and (*h*_*t*−1_) are the LSTM's present and previous states, respectively. To perform the calculations, we need Equations (1–6). Finally, the “$\vec{h}$” hidden state is computed (see Figure 4) by the first hidden layer (forward pass LSTM).

#### 2nd Hidden Layer

**Figure 5:**
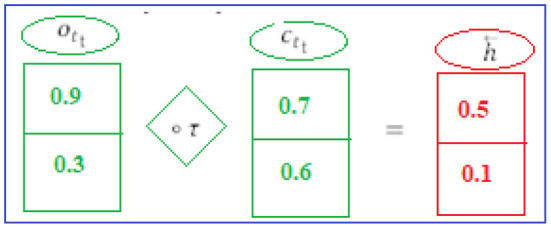
2nd hidden layer.

The backward phase LSTM is composed (see Figure 5) of the current input (*i*_*t*_) and the future state (*h*_*t*+1_). The Equations (7–12) are used to do the computations. The final step is to employ the following layer to generate the hidden state $\overset{⃖}{h}$ (backward phase LSTM).

#### BISLTM Outcome

**Figure 6:**
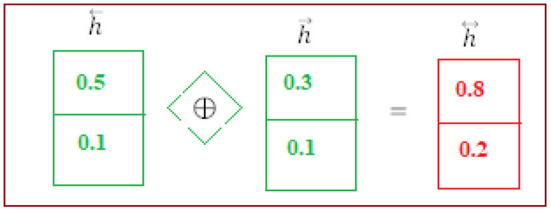
BiSLTM outcome.

Combining forward “$\vec{h}$” and backward “$\overset{⃖}{h}$” from the neural network (see Figure 6), we arrive at the “$\overset{⃡}{h}$” that make up BISLTM's final state (5).

#### Prediction of Hepatitis Patient Survivability

In order to find out how probable each of the tags “D1” and “D2” is, we used the SoftMax technique. The following is an example of how the overall input was calculated using Equation (13).

*For* Hepatitis_patient_ survivability = yes*, the class label for decision attribute 1 is “D1.”*

$$\begin{array}{l} d_{1}=l_{1}\times w_{2}+l_{2}\times w_{2}+b\\ d1=0.6\;\text{x }0.8+\;0.7\;\text{x }0.2+0.5\\ d_{1}=0.48+0.14+0.5=1.12\; \end{array}$$

*For* Hepatitis_patient_ survivability = no*, the class label for decision attribute 2 is “D2.”*

$$\begin{array}{l} d_{2}=l_{1}\times w_{2}+l_{2}\times w_{2}+b\\ d2=0.5\;\text{x }0.8+0.3\;\text{x }0.2+0.5\;\\ d2=0.4+0.06+0.5=0.96\; \end{array}$$

Each target class (D1 and D2) is assigned a probability of success using the SoftMax algorithm (14).

$$\begin{array}{l} softmax\;\left(d_{1}\right)=\frac{\exp^{d_{1}}}{\sum_{i=1}^{n}\exp\left(d_{1}\right)}\\ softmax\;\left(d_{1}\right)=\frac{\exp^{1.12}}{\exp^{1.12}+\exp^{0.96}}\\ softmax\;\left(d_{1}\right)=\frac{3.065\;}{3.065+2.612}=\frac{3.065\;}{5.677}=0.540\; \end{array}$$

Similarly, the SoftMax function was developed for the second class of hepatitis patient survival predictions.

$$\begin{array}{l} softmax\;\left(d_{2}\right)=\frac{\exp^{d_{2}}}{\sum_{i=1}^{n}\exp\left(d_{2}\right)}\\ softmax\;\left(d_{2}\right)=\frac{\exp^{0.96}}{\exp^{1.12}+\exp^{0.96}}\\ softmax\;\left(d_{2}\right)=\frac{2.612\;}{4.305+2.013}=\frac{2.612\;}{5.677}=0.460\; \end{array}$$

**Figure 7:**
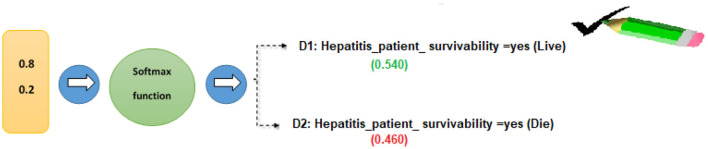
Hepatitis patient survivability classification using the softmax function.

Class D1: Hepatitis patient survivability = yes (live) had the highest likelihood in our calculations (0.681). Based on these data, we may estimate the patient's likelihood of survival as “D1” (live) (Figure 7).

### User Interface

In order to assist in the prediction of hepatitis, the Keras package (3) incorporates a Python user interface. Specialists and other practitioners can use hepatitis prognosis software to better diagnose hepatitis. In order to make the content more accessible and easier to understand, it has been broken down into smaller chunks. In the software's body, there are three main parts: data acquisition, categorization, and hepatitis detection.

**Figure 8:**
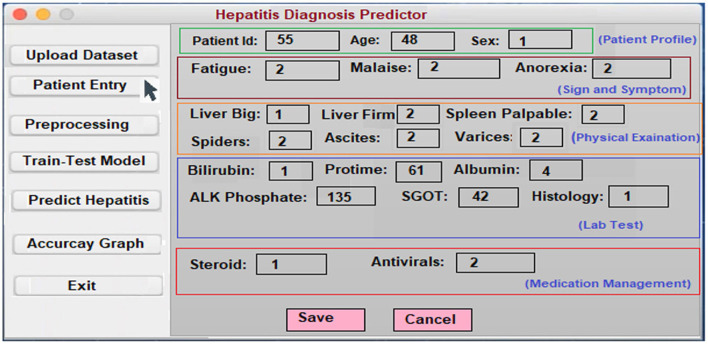
Patient entry form.

**Figure 9:**
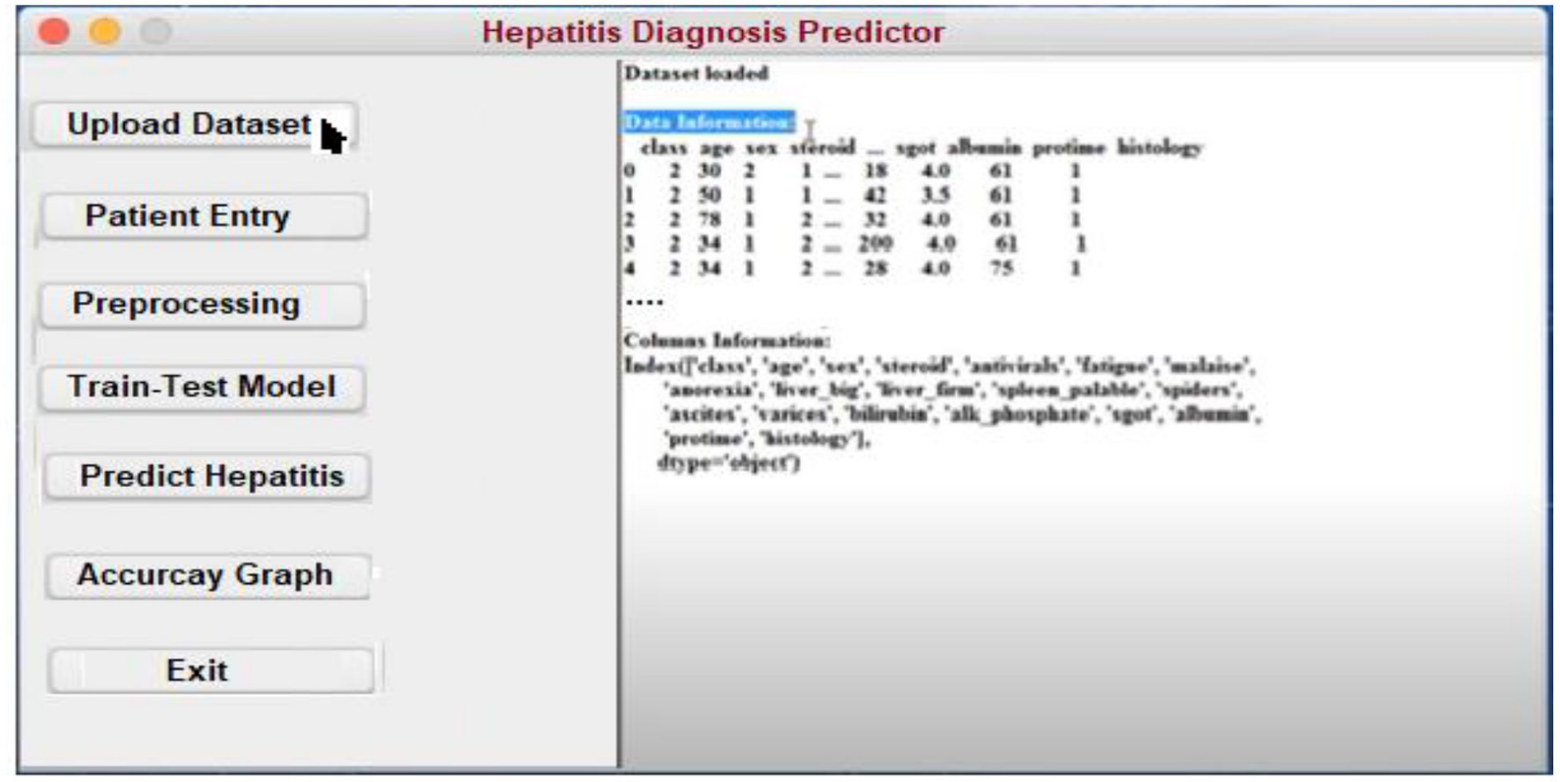
Hepatitis prediction data input form.

**Figure 10:**
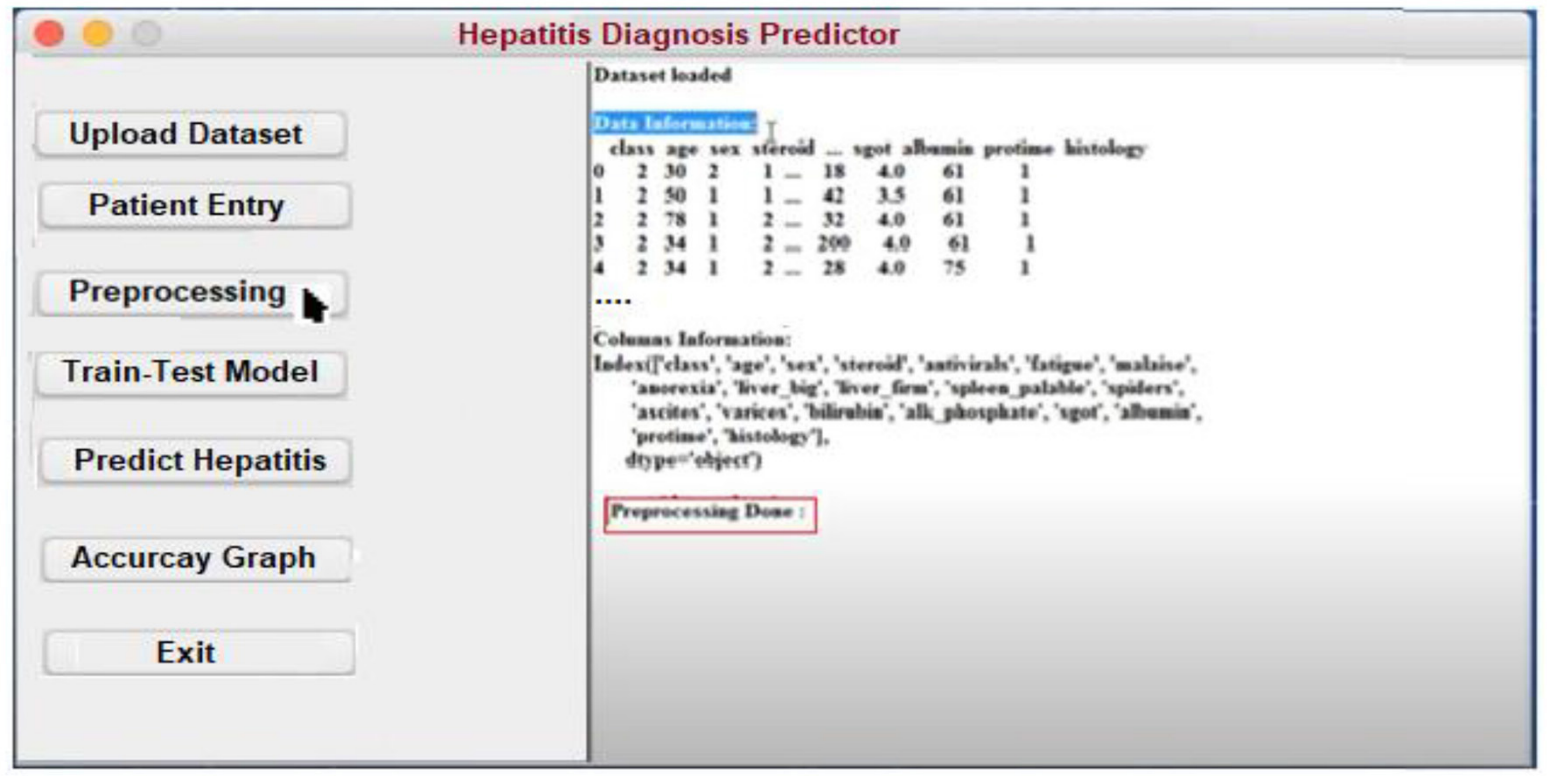
Preprocessing form for Hepatitis prediction.

**Figure 11:**
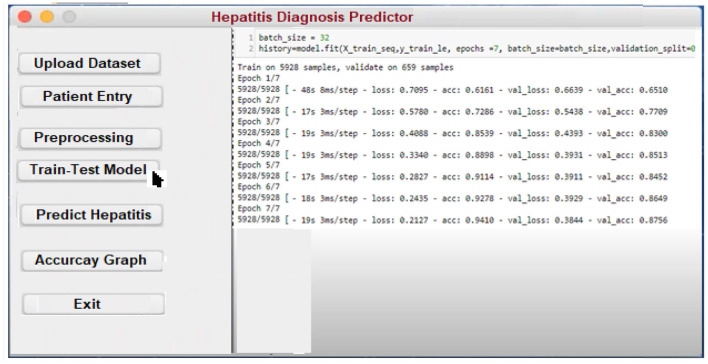
Model training interface.

**Figure 12:**
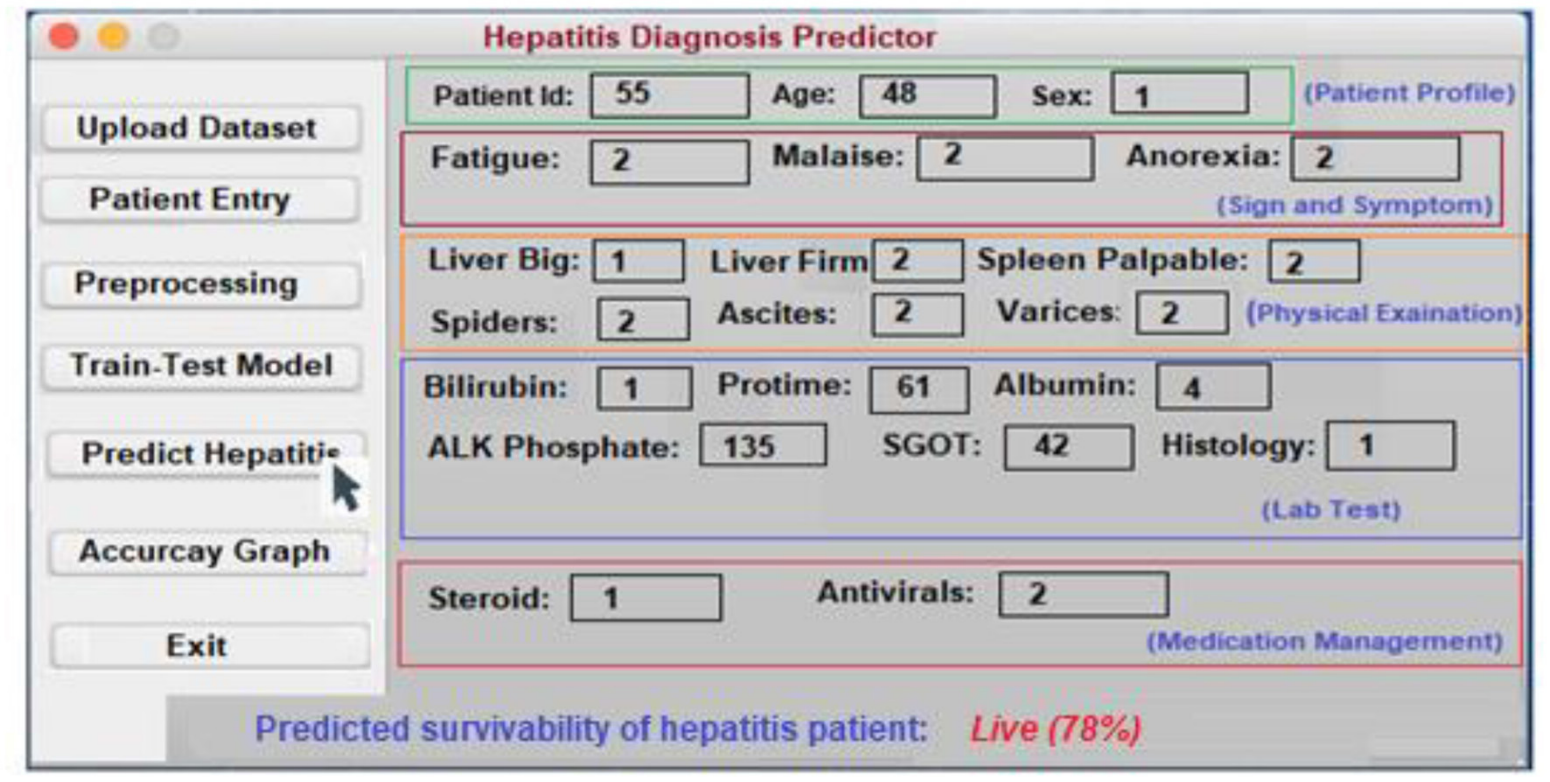
The interface for predicting hepatitis.

- **Patient Entry:** A patient's medical history is required for this section. Each patient in the database has a new case patient identifier created from the improved and more recent information. The hepatitis prediction system utilizes a patient's medical history to generate educated guesses about their condition. As soon as the necessary information is entered into the software, a patient specific identity is generated. Using this identifier, each patient's specific condition may be diagnosed and followed. A patient case entry is shown in Figure 8 on the interface that has been used to gather and evaluate the data. Previous assertions are applied to the data when it is handled on the system's server. It is because of this that we have a design that can be tailored to the needs of every unique patient.
- **Uploading the Dataset:** It is possible to train classifiers and develop models using a hepatitis dataset, as shown in Figure 9.
- **Preprocessing:** Data preprocessing yields a classifier and a model that can predict the outcome of hepatitis. On the backend, the data is preprocessed (Figure 10).
- **Model Training and Testing:** The screen below shows what happens when a patient clicks on the ”Model Training“ option. It serves as a training aid by displaying the loaded data. Model training is activated by clicking on the “train-test model” (Figure 11).
- **Predicting survivability of Hepatitis Patient:** To predict hepatitis, all you need to do is enter the necessary patient information and click the “Predict hepatitis” option on the site. The “Update Training Set” button activates an updated training set. After inputting the patient's disease details and clicking on the “predict hepatitis” button, the findings are shown as “Hepatitis_patient_ survivability = yes,” “D2: Hepatitis_patient_ survivability = no,” and a predicted degree of confidence for each option. “Hepatitis_patient_ survivability = yes (Live)” is the possible outcome of the hepatitis illness prediction for the given list of criteria, as shown in Figure 12.

## Experimental Results and Discussion

This section presents the results of a series of experiments aiming to address the questions raised in section Introduction.

### Experiment#1

Based on patient sickness data, we applied the BiLSTM deep learning model to generate hepatitis predictions.

**Figure 13:**
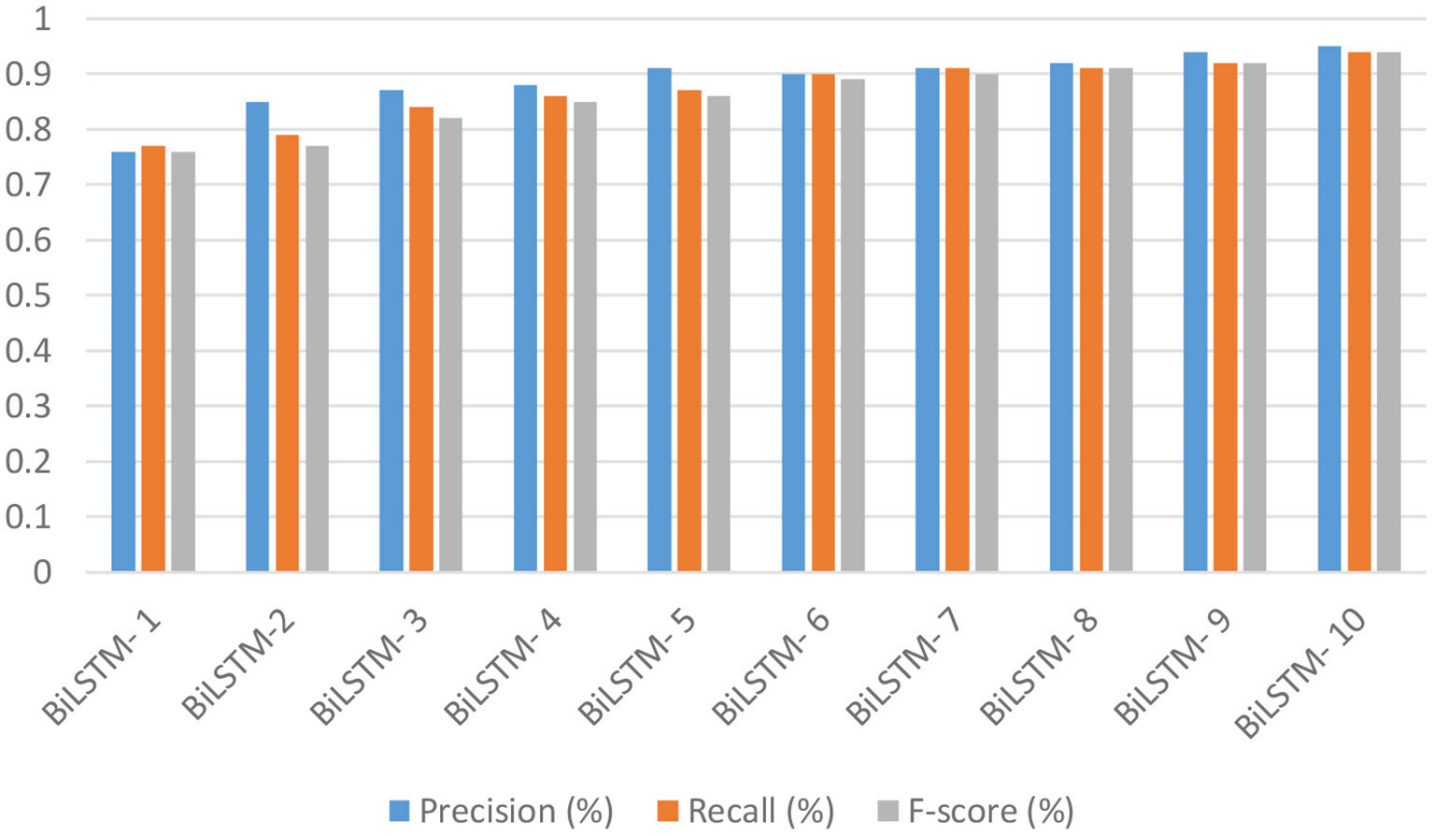
The accuracy, recall, and f1-score of the BiLSTM deep learning models.

Research question # 1 was answered by altering the parameters of one of the most commonly used models for survivability prediction of hepatitis patients, the BiLSTM algorithm. Various additional epochs and filtering techniques were employed as well. Several batch sizes and epochs are included in the algorithm's three hidden layers. As a result of using the SoftMax activation function, there were 62 vocabulary vectors, and the embedding dimension was 128 (8, 16, and 32). A selection of BiLSTM models' accuracy, recall, and F-scores are shown in Figure 13. This filter has an accuracy of 93% when used with the following parameters: filer size of 280, unit size 2, “f1 score” of 94%, recall of 94%, and precision of 94%. Filter number 8 has an accuracy of 95.08%.

The results of 10 BiLSTM experiments with varying parameter values are presented in Table 2. We compared the accuracy of each model against one another. Because it included eight filter sizes, sixteen filters, and ten LSTM units, it had the highest accuracy of all the models tested.

**Table 2:** Accuracy, test loss, and training time of the BiLSTM models.

**Model**	**Test accuracy**	**Test loss**	**Train time (s)**
BiILSTM-1	84%	0.86	20 s
BiLSTM-2	85%	0.83	7 s
BiILSTM-3	86%	1.06	18 s
BiLSTM-4	87%	1.15	16 s
BiLSTM-5	88%	0.88	7 s
BiLSTM-6	90%	0.85	15 s
BiLSTM-7	91%	1.23	12 s
BiLSTM-8	91%	0.78	14 s
BiLSTM-9	92%	0.87	17 s
BiLSTM-10	95%	0.80	12 s

### Experiment#2 With Datasets That Are Balanced and Unbalanced

**Figure 14:**
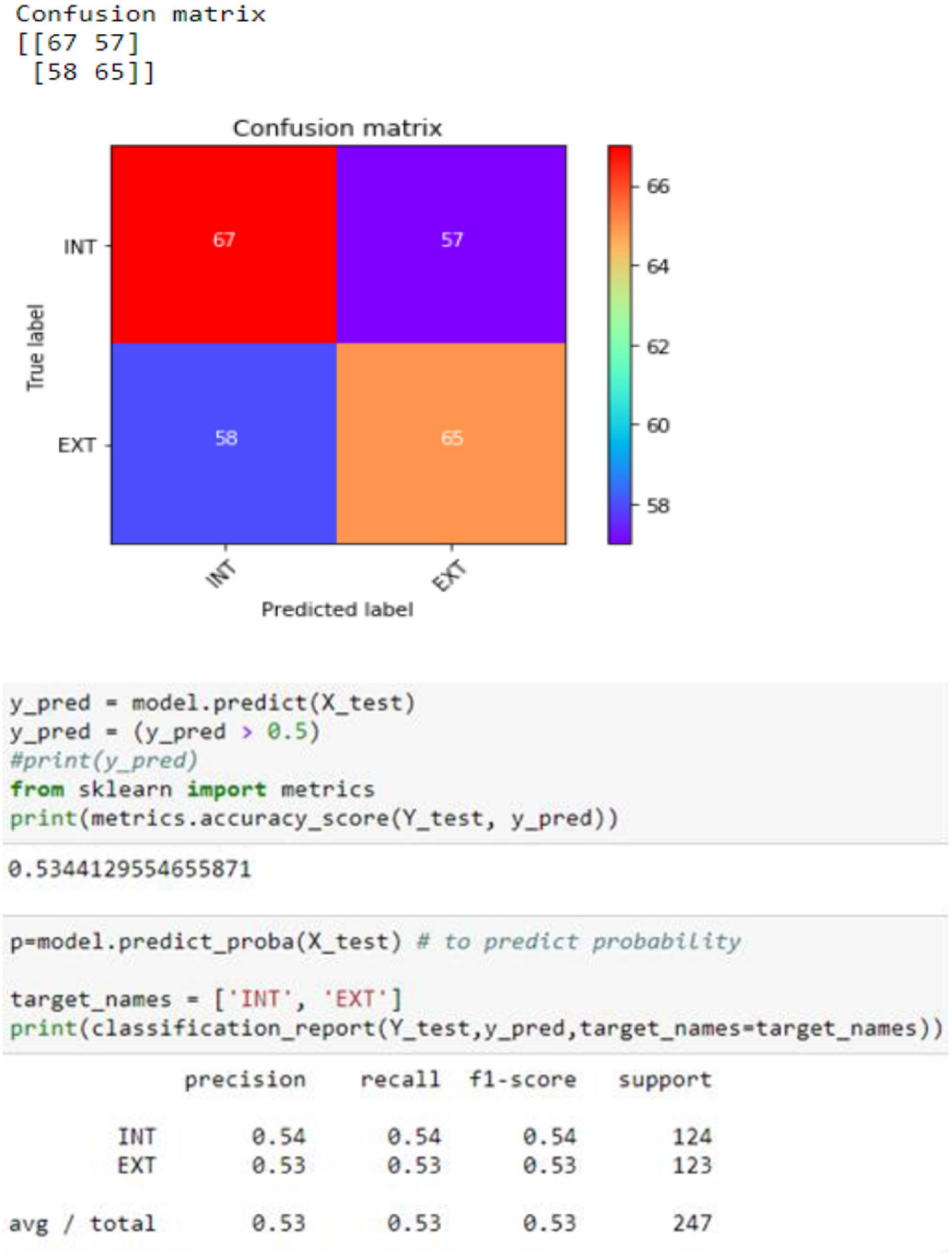
Confusion matrix.

As stated in section 2.0, an imbalanced data classification, randomized training division, and testing set created an uneven class result. It is unbalanced since there are 71 surviving patients and 22 deceased. To equalize the dataset, we used random oversampling. As shown in Figure 14, when data balancing is used instead of not using data balancing, efficiency is significantly increased. By analyzing the empirical values, it is possible that the proposed model may be utilized to properly predict the survivability of hepatitis patients in real-world situations.

### Experiment#3

This experiment is carried out to compare of the BiLSTM model for predicting hepatitis survival with traditional machine learning and deep learning. Conventional machine learning algorithms and deep neural networks were compared to see how the BiLSTM findings for diabetic illness prediction stacked up.

- **ML vs. recommended system (BiLSTM):** Patients' data was used to compare the proposed method (BiLSTM) to other commonly used machine learning methods. Machine learning makes use of feature extraction approaches like TF-IDF and CountVectorizer. Table 3 displays the outcomes of the assessments. A brief summary of the findings is provided in the following paragraphs.
- **BiLSTM vs. SVM:** We utilized the SVM algorithm to compare the proposed BiLSTM model to various machine learning approaches. Table 3 summarizes the findings.
- **BiLSTM vs. KNN:** In this experiment, we compared a typical machine learning classifier with a BiLSTM model. A closer look at Table 3 indicates that KNN classifiers have worse precision, recall, F1-score (0.78), and accuracy (0.77) compared to other classifiers.
- **BiLSTM vs. NB:** In this experiment, the BiLSTM classifier was competing against the Nave Bayes (NB) classification. Table 3 shows that the F1-score (0.74%), recall (0.73%), and precision (0.73%) are all lower than in the previous Table (0.73%).
- **Deep learning vs. suggested method (BiLSTM):** The proposed method is compared to existing deep learning techniques, such as long/short-term memory (LSTM), convolutional neural networks (CNN), and recurrent neural networks (RNN), in order to properly forecast hepatitis based on patient data. Table 4 sums up the findings of the study.
- **LSTM vs. Proposed BiLSTM:** In this study, we evaluated the performance of the BILSMTM model against that of the LSTM model. Table 4 shows that the LSTM model had the lowest precision, recall, F1-score, and accuracy of the four models tested.
- **CNN vs. Proposed BiLSTM:** Our goal was to compare the performance of the BiLSTM approach to that of the CNN model in this particular experiment. For accuracy, precision, recall, F1, and precision, the CNN model behaved poorly in Table 4.
- **RNN vs. Proposed BiLSTM:** We ran this test to see which approach worked best. On the basis of the results in Table 4, it can be concluded that the RNN model's performance on these four metrics is less than ideal.

**Table 3:** Classifiers based on machine learning vs. suggested model (BiLSTM).

**ML modal**	**Accuracy (%)**	**Precision (%)**	**Recall (%)**	**F-score (%)**
SVM	78	79	80	79
KNN	77	78	77	78
NB	74	73	73	73
Proposed (BiLSTM)	95.08	94	93	93

**Table 4:** BiLSTM in comparison to other deep learning models.

**DL model**	**Accuracy (%)**	**Precision (%)**	**Recall (%)**	**F-score (%)**
LSTM	85.14	86	85	85
CNN	85.24	83	84	83
RNN	84.15	82	82	82
Proposed (BiLSTM)	95.08	94	93	93

### Experiment#4

**Figure 15:**
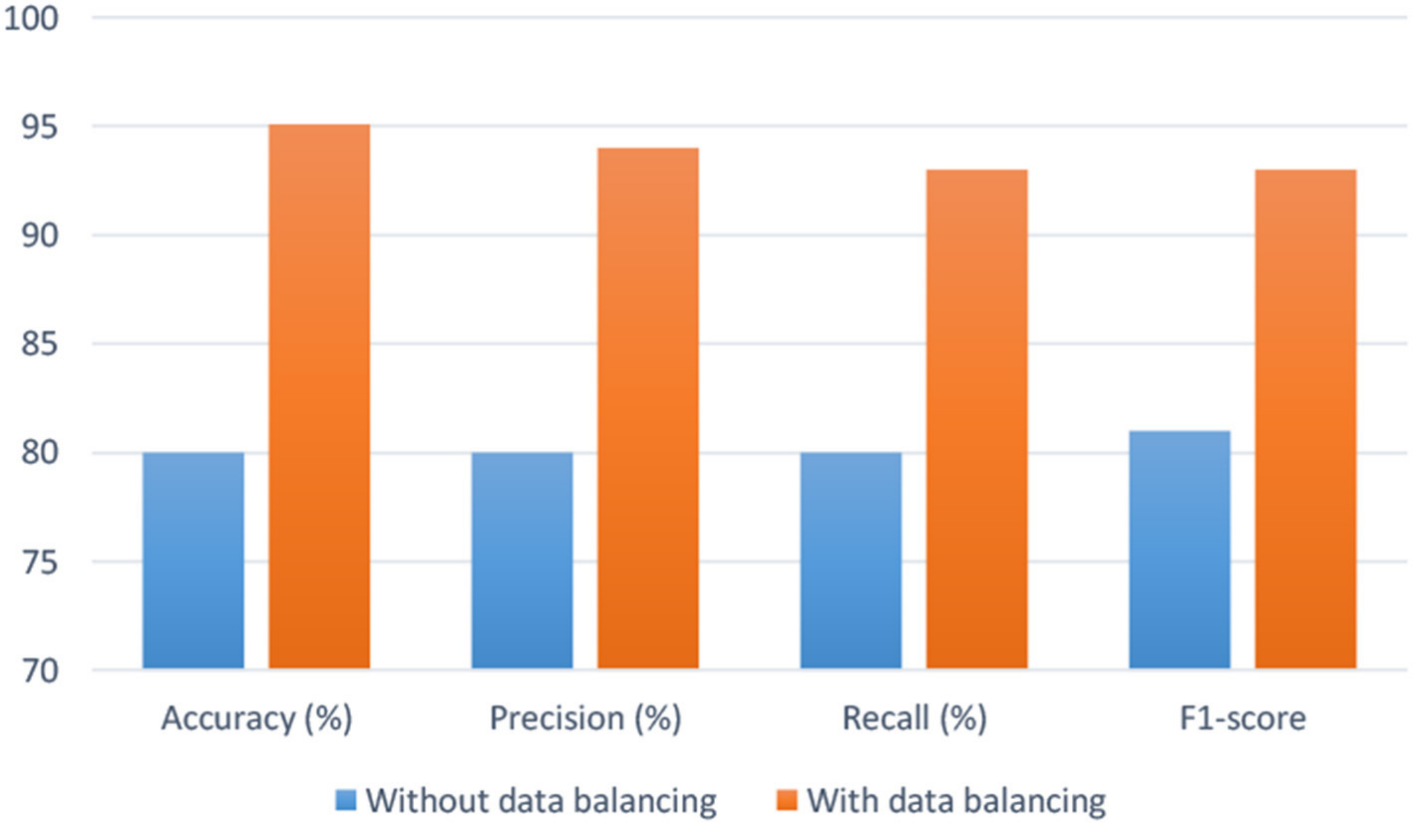
Proposed model's performance with and without balancing data.

In this experiment, the suggested method's efficacy in predicting hepatitis patients is compared to baseline research. In the third research question, we evaluated the suggested BiLSTM model's effectiveness in similar research. To determine how effective the proposed system is, it is contrasted with a variety of other comparing methodologies. According to this study (Figure 15), BiLSTM surpasses a baseline study when compared to the latter. For a variety of reasons, it is difficult to conduct a comprehensive assessment of published methodologies. These approaches were hard to compare because of the variety of datasets.

**Kashif et al**. **(****8****)'s work:** Kashif et al. (8) created an ML-based hepatitis disease prediction method. ML was used to better predict hepatitis using random forest (RF), support vector machine (SVM), and other classifiers

**Akbar et al**. **(****9****)'s work**: Prediction of hepatitis using ML has been proposed. Hepatitis data sets were analyzed using a variety of machine learning methodologies. The performance of a DL model can be improved by using a more effective data balancing method.

**Figure 16:**
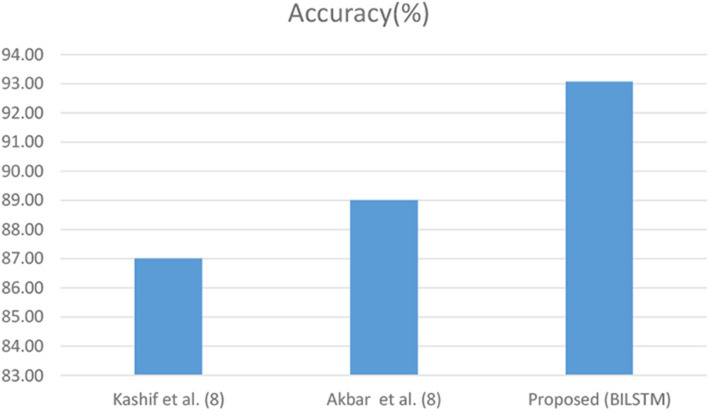
Comparison of other research and the BiLSTM model.

**Proposed work (our model):** The DL-based hepatitis prediction approach uses a deep neural network. Figure 16 shows that the predictor variables (Figure 16) selected have a considerable influence on the anticipated (target) parameter. The combination of data balance and the BiLSTM neural learning model is the most important factor in our achievement in predicting hepatitis illnesses. By using the BiLSTM layer, contextual information can be retained.

### Analyzing Results

A comparison is made between experts' predictions and the prognosis supplied by the suggested strategy, and the performance of the proposed method is evaluated. The processes for the first 12 patients are depicted in Table 5.

**Table 5:** Human expert vs. proposed system prognosis.

**Suspected hepatitis patient**	**Diagnosis by hepatitis expert**	**Prediction by BiLSTM (proposed)**
1	Hepatitis_patient_ survivability = yes	Hepatitis_patient_ survivability = yes
2	Hepatitis_patient_ survivability = yes	Hepatitis_patient_ survivability = yes
3	Hepatitis_patient_ survivability = no	Hepatitis_patient_ survivability = no
4	Hepatitis_patient_ survivability = yes	Hepatitis_patient_ survivability = no
5	Hepatitis_patient_ survivability = yes	Hepatitis_patient_ survivability = yes
6	Hepatitis_patient_ survivability = yes	Hepatitis_patient_ survivability = yes
7	Hepatitis_patient_ survivability = yes	Hepatitis_patient_ survivability = yes
8	Hepatitis_patient_ survivability = no	Hepatitis_patient_ survivability = no
9	Hepatitis_patient_ survivability = yes	Hepatitis_patient_ survivability = yes
10	Hepatitis_patient_ survivability = no	Hepatitis_patient_ survivability = no
11	Hepatitis_patient_ survivability = yes	Hepatitis_patient_ survivability = yes
12	Hepatitis_patient_ survivability = yes	Hepatitis_patient_ survivability = yes

## Conclusion and Future Work

Because of the tremendous expansion in healthcare content, it has become more important to gather and analyze healthcare data in order to discover hepatitis illness in patients. A successful DSS model based on DL was created and put into practice in order to accomplish this. A deep neural network (BiLSTM) is used to predict hepatitis based on data gathering, preprocessing, and validation. Additional experiments were conducted using both balanced and unbalanced data sets. A BiLSTM model was also utilized to predict the chance of having hepatitis in the future. The results are encouraging when compared to prior attempts. The suggested strategy's dependence on embedding rather than on a pre-trained model is an obvious limitation. In the future, the use of pre-trained algorithms like word2vec or Fasttext with hepatitis data sets from different fields (e.g., patient data from different fields) may be looked into.

## Data Availability Statement

The raw data supporting the conclusions of this article will be made available by the authors, without undue reservation.

## Author Contributions

FA and MAs: conceptualization. MAs and FS: methodology. MA-R and JA: software. FA and JA: validation. AK and HN: formal analysis. MR: investigation. MAs: resources. MAl and JA: data curation. MAs: writing—original draft preparation. JA: writing—review and editing. MAs, MS, and JA: visualization. AL: supervision and project administration. FA: funding acquisition. All authors have read and agreed to the published version of the manuscript.

## Funding

The APC was funded by Taif University Researchers Supporting Project Number (TURSP-2020/331), Taif University, Taif, Saudi Arabia.

## Conflict of Interest

The authors declare that the research was conducted in the absence of any commercial or financial relationships that could be construed as a potential conflict of interest. The handling editor declared a past co-authorship with one of the authors MAs.

## Publisher's Note

All claims expressed in this article are solely those of the authors and do not necessarily represent those of their affiliated organizations, or those of the publisher, the editors and the reviewers. Any product that may be evaluated in this article, or claim that may be made by its manufacturer, is not guaranteed or endorsed by the publisher.

## References

[1] Turban E Aronson JE Liang TP MacCarthy RV. Decision Support Systems and Intelligent Systems. Upper Saddle River, NJ: Pearson Prentice-Hall (2005).

[2] Ahmad H Asghar MU Asghar MZ Khan A Mosavi AH. A hybrid deep learning technique for personality trait classification from text. IEEE Access. (2021) 9:146214–32. 10.1109/ACCESS.2021.3121791

[3] Khattak A Habib A Asghar MZ Subhan F Razzak I Habib A. Applying deep neural networks for user intention identification. Soft Comput. (2021) 25:2191–220. 10.1007/s00500-020-05290-z

[4] Rose DC Sutherland WJ Parker C Lobley M Winter M Morris C. Decision support tools for agriculture: towards effective design and delivery. Agric Syst. (2016) 149:165–74. 10.1016/j.agsy.2016.09.009

[5] Shickel B Tighe PJ Bihorac A Rashidi P. Deep EHR: a survey of recent advances in deep learning techniques for electronic health record (EHR) analysis. IEEE J Biomed Health Inform. (2017) 22:1589–604. 10.1109/JBHI.2017.2767063 29989977 PMC6043423

[6] Miotto R Wang F Wang S Jiang X Dudley JT. Deep learning for healthcare: review, opportunities and challenges. Brief Bioinform. (2018) 19:1236–46. 10.1093/bib/bbx044 28481991 PMC6455466

[7] Chicco D Jurman G. An ensemble learning approach for enhanced classification of patients with hepatitis and cirrhosis. IEEE Access. (2021) 9:24485–98. 10.1109/ACCESS.2021.3057196

[8] Kashif AA Bakhtawar B Akhtar A Akhtar S Aziz N Javeid MS. Treatment response prediction in hepatitis C patients using machine learning techniques. Int J Technol Innov Manag. (2021) 1:79–89. 10.54489/ijtim.v1i2.24

[9] Akbar W Wu WP Saleem S Farhan M Saleem MA Javeed A. Development of hepatitis disease detection system by exploiting sparsity in linear support vector machine to improve strength of AdaBoost ensemble model mobile information systems. Personal Commun Technol Smart Space. (2020) 2020:8870240. 10.1155/2020/8870240

[10] Alghazzawi D Bamasaq O Ullah H Asghar MZ. Efficient detection of DDoS attacks using a hybrid deep learning model with improved feature selection. Appl Sci. (2021) 11:11634. 10.3390/app112411634

[11] Panigrahi N Ayus I Jena OP. An expert system-based clinical decision support system for hepatitis-b prediction and diagnosis. Mach Learn Healthcare Appl. (2021) 57–75. 10.1002/9781119792611.ch4

[12] Wicaksno AP Mudiono DRP. Early detection of hepatitis by using certainty factor. In: The First International Conference on Social Science, Humanity, and Public Health (ICOSHIP 2020). Atlantis Press. p. 93–7

[13] Wu C Guo X Li M Shen J Fu X Xie Q. DeepHBV: a deep learning model to predict hepatitis B virus (HBV) integration sites. BMC Ecol Evol. (2021) 21:1–10. 10.1186/s12862-021-01869-8 34233610 PMC8261932

[14] Butt MB Alfayad M Saqib S Khan MA Ahmad M Khan MA. Diagnosing the stage of hepatitis C using machine learning. J Healthc Eng. (2021) 2021:8062410. 10.1155/2021/8062410 35028114 PMC8748759

[15] Orooji A Kermani F. Machine learning based methods for handling imbalanced data in hepatitis diagnosis. Front Health Inform. (2021) 10:57. 10.30699/fhi.v10i1.259

[16] Parisi L RaviChandran N. Syncretic feature selection for machine learning-aided prognostics of hepatitis. Neural Process Lett. (2021) 2021:1–25. 10.1007/s11063-021-10668-7

[17] Wu S Zeng N Sun F Zhou J Wu X Sun Y. HCC prediction models in chronic hepatitis B: a systematic review of 14 models and external validation. Clin Gastroenterol Hepatol. (2021) 19:2499–513. 10.1016/j.cgh.2021.02.040 33667678

[18] UCI Machine Learning Repository: Hepatitis Data Set. (2022). Available online at: https://archive.ics.uci.edu/ml/datasets/hepatitis (accessed January 17, 2022).

[19] Goh M. Predicting Hepatitis Patient Survivability (Uci dataset). Medium. Available online at: https://towardsdatascience.com/predicting-hepatitis-patient-survivability-uci-dataset-71982aa6775d (accessed November 23, 2020)

[20] Khan AS Ahmad H Asghar MZ Saddozai FK Arif A Khalid HA. Personality classification from online text using machine learning approach. Int J Adv Comput Sci Appl. (2020) 11:58. 10.14569/IJACSA.2020.0110358

[21] Alazab M Khan S Krishnan SSR Pham QV Reddy MPK Gadekallu TR. A multidirectional LSTM model for predicting the stability of a smart grid. IEEE Access. (2020) 8:85454–63. 10.1109/ACCESS.2020.2991067

[22] Gadekallu TR Iwendi C Wei C Xin Q. Identification of malnutrition and prediction of BMI from facial images using real-time image processing and machine learning. IET Image Process. (2021) 647–58. 10.1049/ipr2.12222

[23] Bhattacharya S Maddikunta PKR Hakak S Khan WZ Bashir AK Jolfaei A. Antlion re-sampling based deep neural network model for classification of imbalanced multimodal stroke dataset. Multimed Tools Appl. (2020) 1–25. 10.1007/s11042-020-09988-y

